# Quick sequential organ failure assessment versus systemic inflammatory response syndrome criteria for emergency department patients with suspected infection

**DOI:** 10.1038/s41598-021-84743-3

**Published:** 2021-03-05

**Authors:** Atsushi Shiraishi, Satoshi Gando, Toshikazu Abe, Shigeki Kushimoto, Toshihiko Mayumi, Seitaro Fujishima, Akiyoshi Hagiwara, Yasukazu Shiino, Shin-ichiro Shiraishi, Toru Hifumi, Yasuhiro Otomo, Kohji Okamoto, Junichi Sasaki, Kiyotsugu Takuma, Kazuma Yamakawa, Yoshihiro Hanaki, Masahiro Harada, Kazuma Morino

**Affiliations:** 1 Emergency and Trauma Center, Kameda Medical Center, 929, Higashicho, Kamogawa, Chiba 296-8602 Japan; 2 Division of Acute and Critical Care Medicine, Hokkaido University Graduate School of Medicine, Sapporo, Japan; 3 Department of Acute and Critical Care Medicine, Sapporo Higashi Tokushukai Hospital, Sapporo, Japan; 4 Department of General Medicine, Juntendo University, Tokyo, Japan; 5 Health Services Research and Development Center, University of Tsukuba, Tsukuba, Japan; 6 Division of Emergency and Critical Care Medicine, Tohoku University Graduate School of Medicine, Sendai, Japan; 7 Department of Emergency Medicine, School of Medicine, University of Occupational and Environmental Health, Kitakyushu, Japan; 8 Center for General Medicine Education, Keio University School of Medicine, Tokyo, Japan; 9 Center Hospital of the National Center for Global Health and Medicine, Tokyo, Japan; 10 Department of Emergency Medicine, Niizashiki Chuo General Hospital, Niiza, Japan; 11 Department of Acute Medicine, Kawasaki Medical School, Kurashiki, Japan; 12 Department of Emergency and Critical Care Medicine, Aizu Chuo Hospital, Aizuwakamatsu, Japan; 13 Department of Emergency and Critical Care Medicine, St. Luke’s International Hospital, Tokyo, Japan; 14 Trauma and Acute Critical Care Center, Medical Hospital, Tokyo Medical and Dental University, Tokyo, Japan; 15 Department of Surgery, Center for Gastroenterology and Liver Disease, Kitakyushu City Yahata Hospital, Kitakyushu, Japan; 16 Department of Emergency and Critical Care Medicine, Keio University School of Medicine, Tokyo, Japan; 17 Emergency and Critical Care Center, Kawasaki Municipal Kawasaki Hospital, Kawasaki, Japan; 18 Division of Trauma and Surgical Critical Care, Osaka General Medical Center, Osaka, Japan; 19 Department of Emergency and Critical Care Medicine, Japanese Red Cross Nagoya Daiichi Hospital, Nagoya, Japan; 20 Department of Emergency and Critical Care, National Hospital Organization Kumamoto Medical Center, Kumamoto, Japan; 21 Medical Center for Emergency, Yamagata Prefectural Central Hospital, Yamagata, Japan

**Keywords:** Infectious diseases, Medical research

## Abstract

Previous studies have shown inconsistent prognostic accuracy for mortality with both quick sequential organ failure assessment (qSOFA) and the systemic inflammatory response syndrome (SIRS) criteria. We aimed to validate the accuracy of qSOFA and the SIRS criteria for predicting in-hospital mortality in patients with suspected infection in the emergency department. A prospective study was conducted including participants with suspected infection who were hospitalised or died in 34 emergency departments in Japan. Prognostic accuracy of qSOFA and SIRS criteria for in-hospital mortality was assessed by the area under the receiver operating characteristic (AUROC) curve. Of the 1060 participants, 402 (37.9%) and 915 (86.3%) had qSOFA ≥ 2 and SIRS criteria ≥ 2 (given thresholds), respectively, and there were 157 (14.8%) in-hospital deaths. Greater accuracy for in-hospital mortality was shown with qSOFA than with the SIRS criteria (AUROC: 0.64 versus 0.52, difference + 0.13, 95% CI [+ 0.07, + 0.18]). Sensitivity and specificity for predicting in-hospital mortality at the given thresholds were 0.55 and 0.65 based on qSOFA and 0.88 and 0.14 based on SIRS criteria, respectively. To predict in-hospital mortality in patients visiting to the emergency department with suspected infection, qSOFA was demonstrated to be modestly more accurate than the SIRS criteria albeit insufficiently sensitive.

**Clinical Trial Registration:** The study was pre-registered in the University Hospital Medical Information Network Clinical Trials Registry (UMIN000027258).

## Introduction

Sepsis is a health burden in various healthcare settings^1^, especially in emergency departments (EDs). Quick sequential (sepsis-related) organ failure assessment (qSOFA) is a prediction model for mortality following sepsis in patients suspected of having an infection outside the intensive care unit^2,3^. qSOFA was developed and validated in 2016^2^ to replace the systemic inflammatory response syndrome (SIRS) criteria, that were originally designed to determine a systemic inflammatory syndrome. Earlier, SIRS criteria were prerequisite to determine whether patients had sepsis based on the previous definitions of sepsis^4,5^; however, the performance of these criteria was reportedly poor for positive prediction, and insufficient for negative prediction^6,7^.

Of several studies involving patients with suspected infection in EDs to externally validate the prognostic accuracy of qSOFA compared with SIRS criteria in predicting mortality^8–25^ and systematic reviews^26–28^, inconsistency occurred in subsequent external validation results, with better prognostic accuracy of qSOFA for in-hospital mortality than for SIRS criteria. However, most of these studies were retrospective in nature or based on retrospective analyses of prospectively collected data^8,10–25^. These designs had multiple flaws, such as the use of the worst values of predictor variables during ED stay^8–10^ and the use of complete case analysis (excluding subjects with missing variables) for the index tests^9,10,13,14,16–21,23,24^.

Our multicentre prognostic study aimed to prospectively test the hypothesis that qSOFA could predict in-hospital mortality in patients with suspected infection with more accuracy than SIRS criteria using variables obtained at the time when a patient was first suspected of having an infection in the ED.

## Results

### Characteristics of study subjects

This study was discontinued in February 2018 after the number of participants reached 1060 following the recalculation of the required sample size at the interim analysis, which indicated a size of 439 participants. The study participants were mostly older individuals (median age 78, interquartile range [IQR] 68–85 years) with physical impairments (median clinical frailty scale score 4; IQR 3–6) and comorbidities (median Charlson comorbidity index 2; IQR 0–3) (Table 1). Distribution of the site of infection at the ED demonstrated that the most frequent site of infection was the respiratory tract (47.1%), followed by the abdomen (18.7%) and the urinary tract (14.2%).

**Table 1:** Baseline characteristics of the cohort before and after multiple imputation.

Variables	Before multiple imputation		After multiple imputation	
	**Value**	**Missing, %**	**Value**	**Missing, %**
N	1060		1060	
Age^a^, years	78 (68, 85)	0.0	78 (68, 85)	0.0
Male sex^b^	633 (59.7)	0.0	633 (59.7)	0.0
Clinical frailty scale score^a^	4 (3, 6)	0.4	4 (3, 6)	0.0
Charlson comorbidity index^a^	2 (0, 3)	0.0	2 (0, 3)	0.0
**Site of infection**^b^		0.0		0.0
Respiratory tract	499 (47.1)		499 (47.1)	
Urinary tract	151 (14.2)		151 (14.2)	
Abdomen	198 (18.7)		198 (18.7)	
Central nervous system	14 (1.3)		14 (1.3)	
Skin and soft tissue	48 (4.5)		48 (4.5)	
Bone and joint	9 (0.8)		9 (0.8)	
Wounds	4 (0.4)		4 (0.4)	
Catheter	3 (0.3)		3 (0.3)	
Endocardium	6 (0.6)		6 (0.6)	
Implant	2 (0.2)		2 (0.2)	
Other	57 (5.4)		57 (5.4)	
Unknown	69 (6.5)		69 (6.5)	
Respiratory rate, 1/min^a^	22 (18, 28)	0.8	22 (18, 28)	0.0
Systolic blood pressure, mmHg^a^	126 (105, 149)	0.0	126 (105, 149)	0.0
Heart rate, 1/min^a^	98 (84, 113)	0.0	98 (84, 113)	0.0
Glasgow coma scale score^a^	14 (13, 15)	0.0	14 (13, 15)	0.0
Body temperature, °C^a^	37.5 (36.7, 38.5)	0.2	37.5 (36.7, 38.5)	0.0
**White blood cell count, /µL**^a^	10,950 (7500, 14,920)	0.0	10,950 (7500, 14,920)	0.0
Stab cell count, %^a^	69 (11, 88)	66.8	69 (12, 88)	0.0
**Blood gas analysis**^**a**^				
Lactate level, mmol/L	1.9 (1.3, 3.2)	9.3	1.9 (1.3, 3.1)	0.0
Carbon dioxide level, mmHg^a^	37.2 (31.1, 44.1)	9.6	37.0 (31.1, 44.0)	0.0

Data for all variables were obtained at the emergency department at the time when infection was suspected.

^*a*^Numeric variables are reported as median and 25th–75th percentile after pooling the values across the multiply imputed datasets.

^*b*^Nominal variables are displayed as number and percentages after averaging the counts across the multiply imputed datasets and rounding into integers.

### Main results

**Figure 1:**
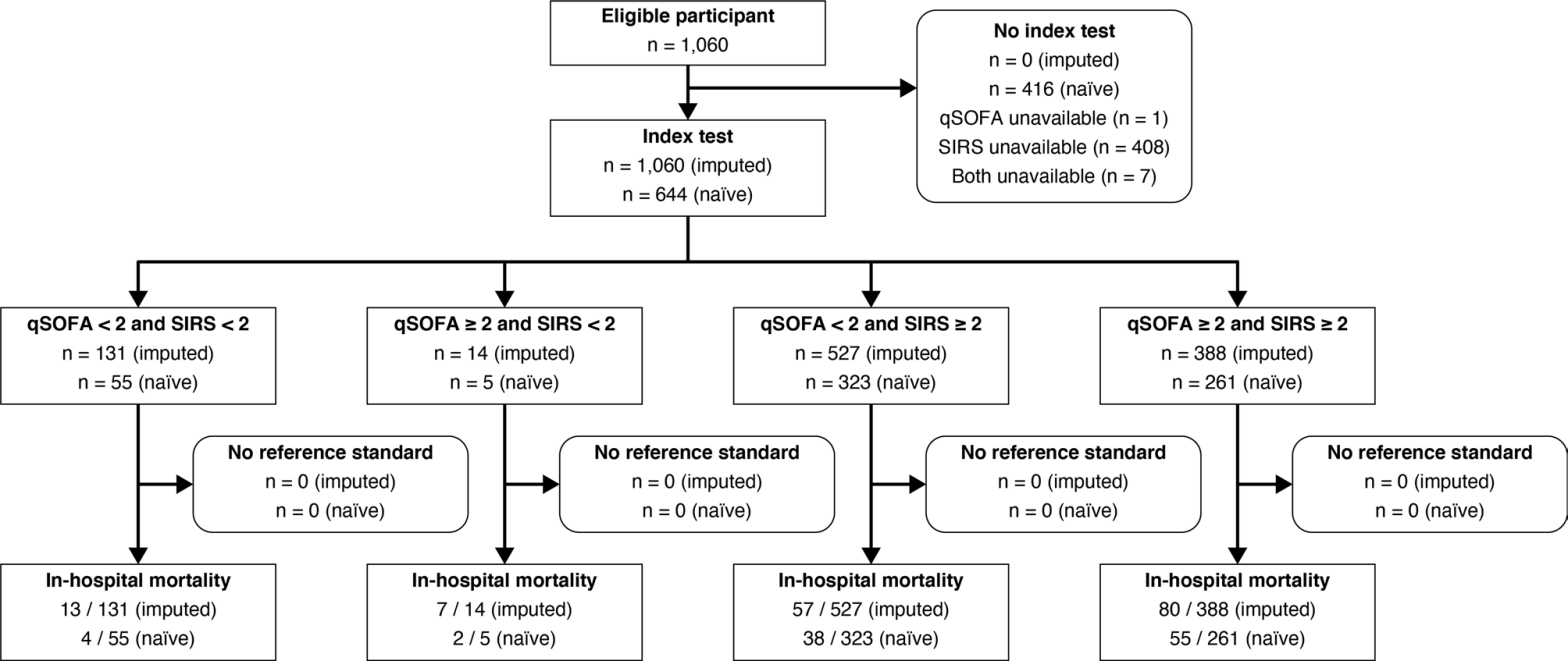
Participant selection tree. All participants recruited in the current study were divided into four groups according to the positivity of the tested scores. The multiply imputed study population and naïve (not imputed) population were included in the primary analysis and sensitivity analysis, respectively. *qSOFA* quick sequential organ failure assessment, *SIRS* systemic inflammatory response syndrome.

**Figure 2:**
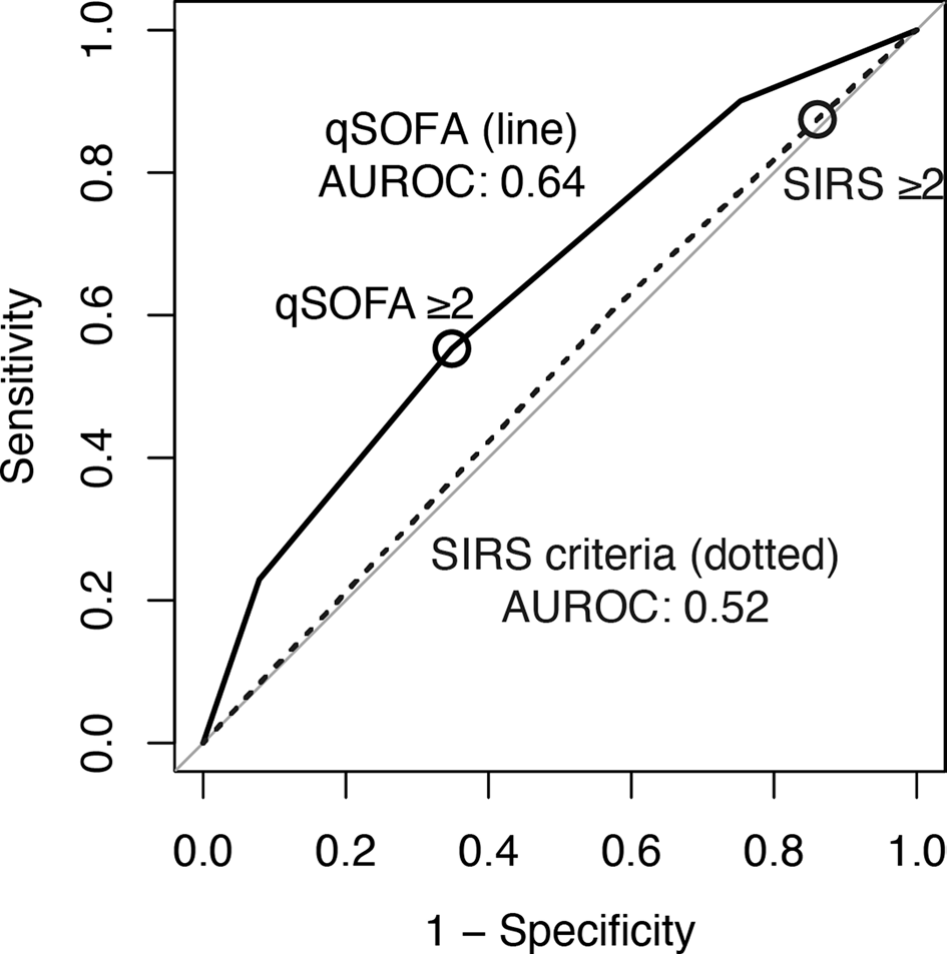
Prediction of in-hospital mortality using the tested scores. Receiver operating characteristics analysis and the prediction of in-hospital mortality using the tested scores (line and dotted line) with the given threshold (circle). *qSOFA* quick sequential organ failure assessment, *SIRS* systemic inflammatory response syndrome, *AUROC* area under the receiver operating characteristic curve.

Missing data for qSOFA, SIRS criteria, and both were found in 1 (0.1%), 408 (38.5%), and 7 (0.7%) participants, respectively (Fig. 1). No missing in-hospital mortality data were reported. In the multiply imputed population, 402 (37.9%) and 915 (86.3%) participants met the thresholds for qSOFA ≥ 2 and SIRS criteria ≥ 2, respectively. A total of 157 (14.8%) participants died in the participating hospitals. The primary analysis demonstrated greater diagnostic accuracy for in-hospital mortality with qSOFA than with SIRS criteria (area under the receiver operating characteristic [AUROC] curve 0.64 versus 0.52, difference + 0.13 95% confidence interval (CI) [+ 0.07, + 0.18]) (Table 2, Fig. 2). Sensitivity and specificity to predict in-hospital mortality at the given thresholds (qSOFA ≥ 2 and SIRS criteria ≥ 2) were 0.55 and 0.65 with qSOFA and 0.88 and 0.14 with SIRS criteria, respectively. The secondary analysis also demonstrated a positive net reclassification improvement (NRI) between qSOFA and SIRS criteria (+ 0.39 95% CI [+ 0.15, + 0.57]).

**Table 2:** Comparison between qSOFA and SIRS criteria prognostic accuracy in predicting in-hospital mortality.

	qSOFA	SIRS	Difference (bootstrap 95% CI)
**Primary analysis**			
Area under the receiver operating characteristics curve	0.64	0.52	+ 0.13 (+ 0.07, + 0.18)
**Secondary analysis**			
Net reclassification improvement analysis			+ 0.39 (+ 0.15, 0.57)

Bootstrap estimation repeated 20,000 times (800 times per imputed dataset), to compute point estimation, and relevant 95% CI.

*95% CI*, 95% confidence interval, *qSOFA* quick sequential organ failure assessment, *SIRS* systemic inflammatory response syndrome, *CI* confidence interval.

**Figure 3:**
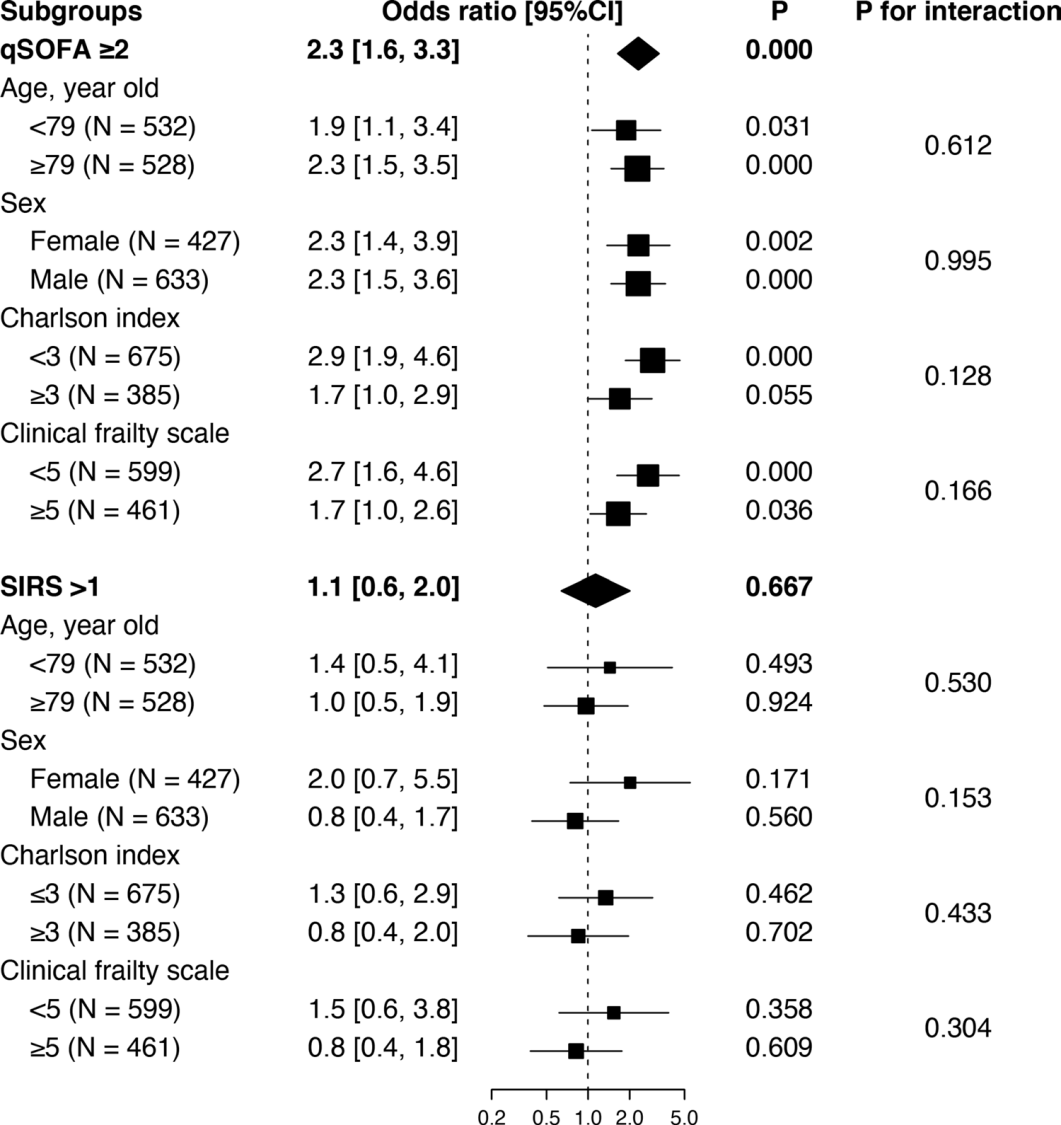
Explanatory subgroup analysis. Study participants were dichotomised according to age (median), sex (female or male), Charlson index (median), and clinical frailty scale score (median) and were subjected to subgroup analysis. The association between positivity of the tested scores (qSOFA ≥ 2 and SIRS criteria ≥ 2) and in-hospital mortality was indicated as an odds ratio with its 95% confidence interval (95% CI). *qSOFA* quick sequential (sepsis) organ failure assessment, *SIRS* systemic inflammatory response syndrome, *95% CI* 95% confidence interval.

Sensitivity analysis of the naive dataset similarly demonstrated greater diagnostic accuracy for in-hospital mortality with qSOFA than with SIRS criteria (AUROC 0.64 versus 0.54, difference + 0.10 95% CI [+ 0.03, + 0.17]), where the sensitivity and specificity to predict in-hospital mortality at the given thresholds were 0.58 and 0.62 with qSOFA and 0.94 and 0.10 with SIRS criteria, respectively. Subgroup analyses did not demonstrate significant interactions with age, sex, comorbidities, and frailty (Fig. 3).

## Discussion

A clinical prediction score is a mathematical model used to estimate the probability of a future event in patients with specific medical conditions. It is essential for a clinical prediction score in the ED to not only deliver a good prediction of the outcome but also provide fewer instances of missing data. The current study demonstrated that the simple qSOFA could provide a better prognostic value as well as better reclassification for in-hospital mortality in patients with suspected infection in the ED, with less frequent cases of missing data than with the SIRS criteria. However, prognostic accuracy of qSOFA (AUROC of 0.64) for mortality is nevertheless insufficient, especially in terms of sensitivity (0.55). Heterogeneity of patients' baseline characteristics, including age, gender, comorbidity index, and frailty score, did not significantly interact with the prognostic ability of both qSOFA and SIRS criteria.

Studies comparing the prognostic accuracy of qSOFA and SIRS criteria in ED settings, except for a single prospective study^9^, have mostly been either purely retrospective in nature^8,12,13,15–21,23–25^ or have been retrospective studies based on prospectively collected data^10,11,14,22^. These studies had several flaws, including acquisition of predictor variables in wide time windows^8–10^ and/or the lack of assessment of computability of missing values^9,10,13,14,16–21,23,24^.

Practically, the qSOFA and SIRS criteria may be applied at the time of the initial evaluation after the patient’s visit to the ED. However, that several studies acquired the predictor variables within a wide time window meant that they could have obtained the worst values during the ED visits, which could bias the prognostic analyses through multiple measurements^8–10^. Use of the worst predicted values obtained with multiple measurements over time could lead to increased and decreased number of participants with positive and negative test results, respectively. Such results might improve the sensitivity and negative predictive value (fewer false negative) or worsen the specificity and positive predictive value (fewer true positive)^15,29^. Furthermore, wide time windows may narrow the time between the prediction and outcome, which can lead to an apparent improvement in the positive prediction result^30^. Finally, prolonged delay in the estimation of the score in the ED is inappropriate in the clinical settings. To avoid such biases and any others arising from multiple measurements, the current study used single baseline values obtained at the time the infection was first suspected.

Computation of qSOFA requires three variables that are readily available at the bedside (respiratory rate, systolic blood pressure, and the Glasgow coma scale [GCS] score). Thus, the score can be rapidly calculated, and the frequency of missing values is low. In contrast, computation of SIRS criteria is complicated and time consuming, and there is a greater likelihood of missing data as six variables consisting of bedside information (respiratory rate, heart rate, and body temperature) and laboratory data (partial carbon dioxide pressure, white blood cell count, and stab cell percentage) are required. In the current study, stab cell count data were missing for almost half of the study population, which led to an increase in the number of incomputable points (39%) and cases with undetermined positivity (12%) of SIRS criteria. However, for qSOFA, these percentages were 1% and 0%, respectively. Furthermore, the large number of missing stab cell count data also led to selection bias if patients with a score of 1 point for normal white blood cell count who lacked stab cell count data were excluded. The current study estimated the prevalence of missing scores and compared the score performance based on both multiply imputed data and naive data, unlike previous studies, which did not sufficiently assess the missing scores^9,10,13,14,16–21,23,24^.

To predict in-hospital mortality in ED patients with suspected infection, qSOFA was generally better and more specific, whereas SIRS criteria were generally worse and more sensitive based on the systematic reviews^26–28^. However, a prominent inconsistency in the estimation of these diagnostic indices has been observed in previous ED studies^8–25^. As discussed, biases in the acquisition of multiple predictor variables and statistical approaches, which ignored missing values, might have led to differences in the estimation of specificity and sensitivity indices for these two methods. Furthermore, even in a prospective study, the authors retrospectively excluded patients with suspected infection at baseline who were later diagnosed with a non-infectious disease^9^. This retrospective exclusion might have led to the apparent improvement in diagnostic accuracy, similar to that in the per-protocol design^9^.

The strength of the current study is its prospective design, which eliminates possible biases in relation to multiple measurements, missing values, and per-protocol analysis. However, there are several limitations to the current study, which should be addressed in the future. First, this was a single national study in a developed country, which limits the generalisability of its results to other patients worldwide with suspected infection. In particular, the study participants were frail older patients with other comorbidities, which might make the results of our study inapplicable to younger populations. Second, even though this was a prospective study, a large percentage of missing stab cell result values prevented a realistic estimation for the SIRS criteria and required the use of multiple imputation. However, we believe the results could reflect those from a real-world setting.

## Conclusions

This prospective, multi-centre study conducted for the external validation of qSOFA and SIRS criteria demonstrated that for patients in the ED who had suspected infection, qSOFA had modestly better prognostic accuracy in predicting in-hospital mortality albeit inadequate in sensitivity, and improved reclassification.

## Methods

### Study design and setting

In 2016, we designed the Sepsis Prognostication in Intensive Care Unit and Emergency Room (SPICE) study, a prospective observational study that consisted of two sub-studies—the SPICE-ER and SPICE-ICU—which were based in the ED and the intensive care unit, respectively. The current study is a primary study of the main SPICE-ER study, which involved a prospective prognostic analysis comparing qSOFA and SIRS criteria and externally validating the prognostic accuracy of these tools for in-hospital mortality among patients in the ED with suspected infection. The design and reporting of the study adheres to the Transparent Reporting of a Multivariable Prediction Model for Individual Prognosis or Diagnosis (TRIPOD) guidelines^31^. The study included 34 EDs from 6 secondary and 28 tertiary emergency care centres and was conducted between December 2017 and February 2018. All procedures in these studies that involved human participants were performed in accordance with the ethical standards of the institutional and/or national research committee and with the 1964 Declaration of Helsinki and its later amendments or comparable ethical standards. The study protocol for this observational study, including a waiver of the informed consent requirement, was first approved by the institutional review board of Hokkaido University (approval number 016-0385) and subsequently by the ethics committees of all participating hospitals.

### Selection of participants

For this study, we included patients who visited the EDs of the participating hospitals; who were suspected of having an infection by the emergency physicians; who received any kind of antibiotics, underwent any fluid culture test, or underwent imaging for the detection of infection sites during ED stay; and who were hospitalised or died in the ED. Patients were excluded if they were transferred to another hospital without hospitalisation at the participating hospital.

### Measurements

Index tests (qSOFA and SIRS criteria) were assessed using the data collected on clinical variables at the time when the infection was first suspected. Both qSOFA and SIRS criteria tested positive at a score ≥ 2 point based on the original definitions of sepsis^2,4^. GCS scores < 15 were used to satisfy the altered mental status criteria in qSOFA^2^. The reference standard for the study was in-hospital mortality. The application of multiple imputation on all the study variables enabled 100% calculation of the study index tests and 100% assessment of associations between the index tests and the study outcome.

### Outcomes

The baseline characteristics examined included the patients’ statuses before index infection, i.e., age, sex, clinical frailty scale score^32^, and Charlson comorbidity index^33^, as well as the clinical data obtained at the ED, i.e., from the suspected site of infection and on the physiological status (respiratory rate, heart rate, systolic blood pressure, the GCS score, body temperature, white blood cell count, stab cell percentage, lactate level, and partial pressure of carbon dioxide in the blood gas analysis). The suspected sites of infection were classified into 12 regions: the respiratory tract, urinary tract, abdomen, central nervous system, skin and soft tissue, bones and joints, wounds, intravascular catheter, endocardium, any kind of implant aside intravascular catheter, other regions, and unknown origin. The study outcome was defined as in-hospital mortality during ED stay or hospitalisation. All study data from the participating hospitals were entered electronically into the data capture server provided by the University Hospital Medical Information Network Internet Data and Information Center for Medical Research.

### Statistical analysis

The statistical parameters required for estimating the sample size in this study were not fully available from previous publications; therefore, this study employed an adaptive design for sample size estimation. Initial sample size estimation was done using the receiver operating characteristics (ROC) curve power calculation method based on the hypothesis that SIRS criteria significantly predicted in-hospital mortality^34^. Parameters needed for the initial sample size estimation included AUROC curve for in-hospital mortality with an SIRS criteria score of 0.64, probability of in-hospital mortality of 0.04^2^, power of 0.8, and significance level of 0.05. The required initial sample size was estimated at 807 participants but was modified to 900 participants considering the decline in statistical power owing to missing values. Interim analysis was pre-planned to determine the final study sample size based on two ROC curve power calculations to detect differences in the AUROCs of tested scores when the number of the study participants exceeded the initial sample size^35^. The study was to be discontinued after the number of participants exceeded the estimated sample size at the interim analysis. Additionally, the study was also to be discontinued if the estimated sample size exceeded the upper limit of 2000 participants.

To compensate for the missing values, mainly in laboratory variables, multiple imputation by chained equations, which generated 25 multiply imputed datasets with 20 iterations of calculations, was used^36^.

The statistical analysis plan consisted of the comparison of AUROC (primary analysis) and NRI analysis^37^ (secondary analysis). Integration of the point estimations with 95% confidence intervals across each analysis on the multiply imputed dataset was based on bootstrapping. It was repeated 800 times per multiply imputed dataset to a total of 20,000 times.

In consideration of possible inconsistencies in the results before and after multiple imputation, a sensitivity analysis was conducted to assess the robustness of the results from primary analysis, using the naive dataset prior to multiple imputation instead of the multiply imputed datasets.

It was assumed that heterogeneity in the baseline characteristics of the participants might interact with the predicted scores for in-hospital mortality. A post-hoc subgroup analysis, dichotomised by age, sex, the clinical frailty scale score^32^, and Charlson comorbidity index^33^ was used to assess the association between positivity of the tested scores and in-hospital mortality, which was reported as an odds ratio.

All statistical analyses were performed using R version 3.5.2 statistical software (The R Foundation for Statistical Computing, Vienna, Austria).

### Ethics approval and consent to participate

The study was conducted after obtaining approval from the ethics committees of all participating hospitals. All procedures in these studies that involved human participants were performed in accordance with the ethical standards of the institutional and/or national research committee and with the 1964 Declaration of Helsinki and its later amendments or comparable ethical standards. The requirement for informed consent was waived by the ethics committees because of the observational nature of the study.

## Data Availability

The datasets used and/or analysed during the current study are available from the corresponding author on reasonable request.
